# Effects of Dietary Recombinant Irisin on Growth Performance, Serum Antioxidant and Immune Indices, and Cecal Microbiota of Broilers

**DOI:** 10.3390/vetsci13080811

**Published:** 2026-08-15

**Authors:** Pei Wen, Haiyang Wu, Feitao Dang, Jinbo Sun, Hongwei Shi, Yi Yan, Jiayin Lu, Xiaomao Luo, Yanjun Dong, Haidong Wang

**Affiliations:** 1 Shanxi Key Laboratory of Animal Disease Research, Prevention and Control, College of Veterinary Medicine, Shanxi Agricultural University, Jinzhong 030801, China; 252004@stu.sxau.edu.cn (P.W.); 202420387@stu.sxau.edu.cn (H.W.); dangfeitao@sxau.edu.cn (F.D.); 20233892@stu.sxau.edu.cn (J.S.); 20231061@stu.sxau.edu.cn (H.S.); yanyi@sxau.edu.cn (Y.Y.); jiayinlu@sxau.edu.cn (J.L.); xmluo@sxau.edu.cn (X.L.); 2 College of Veterinary Medicine, China Agricultural University, Beijing 100091, China; yanjund@cau.edu.cn

**Keywords:** recombinant protein, feed efficiency, carcass yield, oxidative status, immunoglobulins, cecal microbiome

## Abstract

Irisin is a myokine derived from fibronectin type III domain-containing protein 5 (FNDC5) and is involved in energy metabolism, muscle development, and oxidative regulation. Although irisin has been extensively studied in mammals, its potential application in poultry production remains unclear. Enhancing growth performance and physiological resilience is critical for male white-feathered broiler chickens under intensive production systems. This study investigated the effects of dietary irisin supplementation on growth performance and physiological responses in male white-feathered broiler chickens. A total of 180 broilers were randomly allocated to three treatments, with six replicate pens of ten birds per treatment: basal diet and basal diet supplemented with 5 and 10 mg/kg irisin for 42 days. At 42 days, one bird from each replicate pen was sampled for carcass, meat-quality, serum, immune, and microbiota analyses. Dietary irisin supplementation significantly increased final body weight and average daily gain compared with the control group. Feed conversion ratio decreased from 1.51 in the control group to 1.46 in the 5 and 10 mg/kg groups. Carcass evaluation showed higher slaughter yield and breast muscle yield in irisin-supplemented birds, and cooking loss was significantly reduced in both breast and thigh muscles. Serum superoxide dismutase (SOD) activity was elevated in irisin-treated groups. In addition, immunoglobulin Y (IgY) and immunoglobulin M (IgM) concentrations were significantly increased. No significant treatment effects were observed for total antioxidant capacity (T-AOC), catalase (CAT), malondialdehyde (MDA), inflammatory cytokines (IL-6, TNF-α), or immune organ indices (thymus, spleen and bursa of Fabricius). Gut microbiota analysis showed numerical differences in the relative abundance of several major bacterial taxa among treatments. In conclusion, dietary irisin supplementation was associated with improved growth performance and selected carcass, meat-quality, antioxidant, immunoglobulin, and cecal microbiota outcomes in male white-feathered broiler chickens, supporting further investigation of its potential as a functional feed additive in poultry nutrition.

## Simple Summary

Improving growth while maintaining the health of broiler chickens is important for sustainable poultry production. Irisin is a protein involved in energy use, muscle development, and protection against oxidative stress, but its effects in poultry are not well understood. In this study, 180 male white-feathered broilers received a standard diet or diets supplemented with 5 or 10 mg/kg recombinant irisin for 42 days. Irisin supplementation increased final body weight and daily weight gain and improved feed conversion. Carcass evaluation showed higher carcass-related yields and breast muscle yield in irisin-supplemented birds. Cooking loss was significantly reduced in both breast and thigh muscles. It also increased serum superoxide dismutase (SOD) activity and serum immunoglobulin Y (IgY) and immunoglobulin M (IgM) concentrations. In addition, irisin supplementation was associated with numerical differences in the relative abundance of several major bacterial taxa. These findings suggest that dietary irisin may serve as a potential functional feed additive affecting broiler growth, feed efficiency, selected physiological indices, and intestinal microbial composition. Further studies are required to confirm its mechanisms of action, safety, and practical value under commercial production conditions.

## 1. Introduction

Maximizing growth performance and feed efficiency while maintaining flock health is a major objective in modern broiler production [1]. Intensive genetic selection has resulted in white-feathered broilers with rapid growth rates and high breast muscle yield; however, these traits are accompanied by increased metabolic demands and greater susceptibility to nutritional and environmental stressors [2,3]. Under commercial production conditions, oxidative imbalance and impaired immune homeostasis may negatively influence nutrient utilization, growth efficiency, and overall production performance [4]. Therefore, identifying nutritional strategies that enhance antioxidant capacity and immune function represents an important approach for improving broiler productivity.

Irisin, a peptide hormone derived from fibronectin type III domain-containing protein 5 (FNDC5), has attracted considerable attention as a regulator of energy metabolism [5,6]. Initially characterized as an exercise-associated myokine in mammals, irisin has been implicated in regulating mitochondrial function, oxidative metabolism, and metabolic homeostasis [7,8]. In addition, accumulating evidence indicates that irisin may influence antioxidant defense systems and immune-related processes, suggesting its potential application as a bioactive nutritional factor [8].

Although the physiological functions of irisin have been extensively studied in mammalian models, its biological effects and nutritional potential in poultry remain poorly understood [9,10]. Recombinant protein-based feed additives represent a promising strategy for improving animal health and productivity; however, their efficacy must be evaluated under practical feeding conditions [11]. In broilers, it remains unclear whether dietary supplementation with recombinant chicken irisin can influence growth performance, carcass development, meat quality, antioxidant status, immune-related parameters, and intestinal microbial ecology.

The gastrointestinal tract represents a critical interface determining the biological effects of orally administered proteins. Factors including feed processing conditions, gastrointestinal digestion, and intestinal availability may influence the exposure and activity of dietary recombinant proteins [12]. Therefore, comprehensive evaluation of production-related outcomes is necessary to determine whether recombinant irisin supplementation produces measurable physiological benefits in broilers.

In the present study, we investigated the effects of dietary recombinant chicken irisin supplementation on growth performance, feed efficiency, carcass characteristics, meat quality, antioxidant capacity, humoral immune responses, and cecal microbial composition in white-feathered broilers. We hypothesized that dietary irisin supplementation would improve production performance and modulate antioxidant, immune, and gut microbial profiles, thereby providing experimental evidence for its potential use as a functional feed additive in broilers.

## 2. Materials and Methods

### 2.1. Animals and Experimental Design

A total of 180 one-day-old male Arbor Acres (AA) broiler chickens were obtained from Shanxi Daxiang Agriculture and Animal Husbandry Group (Shanxi, China). Upon arrival, birds were individually weighed, and the initial body weight was 39.58 ± 0.22 g (mean ± SD). Birds were then randomly allocated to three dietary treatments in a completely randomized design, with six replicate floor pens per treatment and ten birds per pen. Pens assigned to different treatments were randomly distributed within the poultry house to minimize potential positional effects. Each pen measured 1.2 m × 1.0 m. The treatments consisted of a basal diet without irisin supplementation and the basal diet supplemented with 5 or 10 mg/kg recombinant chicken irisin.

The experimental period lasted for 42 d and was divided into a starter phase (1–21 d) and a grower phase (22–42 d). The basal diets were formulated based on the nutrient requirements of broiler chickens recommended by the National Research Council (NRC, 1994) [13], the Arbor Acres Broiler Management Handbook [14], and the Arbor Acres Broiler Nutrition Specifications [15]. The ingredient composition and calculated nutrient levels of the basal diets are shown in Table 1.

Birds were housed in environmentally controlled floor pens equipped with feeders and nipple drinkers, allowing ad libitum access to feed and water. The room temperature was maintained at approximately 33 °C during the first week and gradually reduced to 22 °C by the end of the third week. A lighting program of 23 h light and 1 h dark was applied during the first week, followed by 18 h light and 6 h dark thereafter. Illumination was provided by overhead 520-nm monochromatic green LED lamps. All management practices were conducted according to standard commercial broiler production guidelines.

At 42 d, after a 12-h feed withdrawal, one clinically healthy bird from each replicate pen (6 males per group; 18 males in total) was selected for blood collection, slaughter, carcass evaluation, meat quality analysis, immune organ measurement, and sample collection. Birds with body weights closest to the mean body weight of their respective pens were selected to minimize variation associated with individual body weight differences.

### 2.2. Diets and Irisin Supplementation

Recombinant chicken irisin was produced and purified in our laboratory. The coding sequence corresponding to the extracellular domain of chicken FNDC5 was cloned into an expression vector and expressed in Escherichia coli. The recombinant protein was purified using nickel–nitrilotriacetic acid affinity chromatography, followed by endotoxin removal. Protein purity was confirmed by SDS-PAGE, and protein concentration was determined using a bicinchoninic acid assay. The purity of recombinant irisin was greater than 95%. The purified protein was lyophilized and stored at −20 °C until use.

Basal diets were prepared in batches, sealed in moisture-resistant bags, and stored under controlled conditions (18–20 °C and 50–60% relative humidity) until use. To minimize potential degradation of irisin during feed preparation and storage, the required amount of lyophilized irisin was freshly dissolved in physiological saline before feeding each day, mixed with a small amount of basal diet to prepare a premix, and then gradually incorporated into the daily ration. The control diet was prepared using the same basal formulation and mixing procedure without irisin supplementation. No antibiotics or growth-promoting additives were included in the experimental diets. All diets were provided in mash form and offered ad libitum throughout the 42-d experimental period.

### 2.3. Measurements

#### 2.3.1. Growth Performance

Growth performance was evaluated using the replicate pen as the experimental unit. Body weight (BW) and feed intake (FI) were recorded on days 1, 21, and 42. Before weighing on days 21 and 42, birds were subjected to a 12-h feed withdrawal. Mortality and culling were monitored and recorded daily throughout the experimental period. Average daily gain (ADG) was calculated as the difference between final and initial BW divided by the number of experimental days. Average daily feed intake (ADFI) was calculated on a bird-day basis as the total feed intake per pen divided by the cumulative number of bird-days during the corresponding period. Feed conversion ratio (FCR) was calculated as total feed intake divided by total body weight gain. If mortality occurred, FCR was adjusted by including the body weight of dead birds in the total weight gain.

#### 2.3.2. Carcass Traits

Carcass evaluation was performed according to the Chinese standard NY/T 823-2004 (Performance Terminology and Measurements for Poultry) [16]. Live body weight was recorded before slaughter. Carcass weight was measured after bleeding and defeathering, and semi-eviscerated and eviscerated weights were determined after removal of internal organs according to the standard procedure. Breast muscle yield was determined by dissecting and weighing the pectoralis major and pectoralis minor muscles from both sides of the chest. Leg muscle yield was determined by dissecting and weighing all thigh and drumstick muscles from both legs. Abdominal fat was excised and weighed. Slaughter yield, semi-eviscerated yield, eviscerated yield, breast muscle yield, leg muscle yield, and abdominal fat percentage were calculated as percentages of live body weight or eviscerated weight.

#### 2.3.3. Meat Quality

Breast and thigh muscle samples were collected from the same birds for meat quality analysis. Muscle pH was measured at 45 min and 24 h postmortem using a calibrated pH meter (Sartorius, Göttingen, Germany), with three measurements averaged per sample. Meat color (L*, a*, and b*) was measured on the exposed surface of intact breast and thigh muscles using a CR-310 colorimeter (Minolta Camera Co., Ltd., Osaka, Japan), with three replicate measurements per sample. Drip loss was determined using approximately 50 g samples trimmed to approximately 5 cm × 1 cm × 1 cm, suspended at 4 °C for 24 h, and calculated as percentage weight loss. Cooking loss was determined using approximately 25 g samples boiled in water for 45 min; samples were removed, held for 15 min, blotted to remove surface moisture, and reweighed. Shear force was measured on the cooked samples using a TA.XT Plus Texture Analyser (Stable Micro Systems, Godalming, UK). After cooking and cooling, samples were trimmed into cores parallel to the longitudinal orientation of muscle fibers, and shear force was determined perpendicular to the direction of muscle fibers. Multiple subsamples were measured for each sample, and the average value was used for statistical analysis. The test speed was set at 2.0 mm/s.

#### 2.3.4. Serum Biochemical Parameters

Approximately 5 mL of blood was collected from the wing vein, allowed to clot at room temperature for 10 min, and centrifuged at 3000× *g* for 15 min. Serum was collected and stored at −80 °C until analysis. Serum levels of total protein (TP; Cat# G0432W), albumin (ALB; Cat# G1208W), glucose (GLU; Cat# G1214W), urea (Cat# G1201W), triglycerides (TG; Cat# G0910W), and total cholesterol (T-CHO; Cat# G0909W) were determined using commercial microplate assay kits (Suzhou Grace Biotechnology Co., Ltd., Suzhou, China) in accordance with the manufacturer’s protocols.

#### 2.3.5. Antioxidant Indices

Serum antioxidant status was evaluated by measuring total antioxidant capacity (T-AOC; G0142W, ABTS method), superoxide dismutase (SOD; G0101W, WST-8 method), catalase (CAT; G0105W), and malondialdehyde (MDA; G0109W) using commercial microplate assay kits (Suzhou Grace Biotechnology, Suzhou, China) according to the manufacturer’s instructions.

#### 2.3.6. Immune Organ Indices

The thymus, spleen, and bursa of Fabricius were excised and weighed immediately after slaughter. Immune organ indices were calculated as organ weight (g) divided by live body weight (kg).

#### 2.3.7. Serum Immunoglobulins and Cytokines

Serum concentrations of immunoglobulin Y (IgY), immunoglobulin M (IgM), interleukin-6 (IL-6; BYHS507592), and tumor necrosis factor-α (TNF-α) were determined using chicken-specific commercial ELISA kits (Nanjing Boyan Biotechnology, Nanjing, China) according to the manufacturer’s instructions.

#### 2.3.8. Gut Microbiota Analysis

Cecal-content samples from five birds per treatment (15 samples in total) were submitted to Majorbio Bio-Pharm Technology Co., Ltd. (Shanghai, China). Samples were aseptically collected, immediately frozen in liquid nitrogen, and stored at −80 °C until analysis. Microbial genomic DNA was extracted using a commercial DNA extraction kit (Quanshijin Biotechnology, Beijing, China). The V3–V4 region of the bacterial 16S rRNA gene was amplified using primers 338F and 806R with TransStart FastPfu DNA Polymerase and sequenced using an Illumina paired-end sequencing platform (Illumina, Inc., San Diego, CA, USA).

Sequencing quality control, read assembly, and primary bioinformatic processing were performed by Majorbio Bio-Pharm Technology Co., Ltd. under its standard quality-control workflow. Paired-end reads were quality-filtered, merged, and denoised using the QIIME 2 workflow to generate amplicon sequence variants (ASVs), with chimeric sequences removed during denoising. The ASV table was rarefied to 36,369 reads per sample before downstream analysis. Alpha diversity, beta diversity, and taxonomic composition at the phylum and genus levels were evaluated.

### 2.4. Statistical Analysis

Data are presented as mean ± SEM. Statistical analyses were performed using IBM SPSS Statistics 20.0 (IBM Corp., Armonk, NY, USA). The replicate pen was considered the experimental unit for growth performance and pen-based measurements, whereas individual birds were used for bird-level measurements. Normality and homogeneity of variance were assessed using the Shapiro–Wilk and Levene’s tests, respectively. Data were analyzed using one-way ANOVA followed by Bonferroni-adjusted pairwise comparisons. Growth-performance variables were considered primary outcomes, whereas carcass traits, meat quality, serum parameters, antioxidant indices, immune measurements, and microbiota-related variables were considered secondary outcomes. Differences were considered statistically significant at *p* < 0.05.

## 3. Results

### 3.1. Growth Performance

As shown in Table 2, dietary irisin supplementation significantly increased final body weight at 42 days (*p* = 0.017). Broilers fed 5 or 10 mg/kg irisin reached 2525.17 and 2526.88 g, respectively, compared with 2415.50 g in the control group. For the overall period (1–42 d), average daily gain was significantly higher in the 5 and 10 mg/kg groups (59.17 and 59.22 g/d) than in the control group (56.57 g/d; *p* = 0.017). Average daily feed intake was also increased (*p* < 0.001), with the highest value observed in the 10 mg/kg group (86.73 g/d). Feed conversion ratio decreased from 1.51 in the control group to 1.46 in both irisin-supplemented groups (*p* = 0.029).

### 3.2. Carcass Traits

As shown in Table 3, dietary irisin supplementation significantly increased slaughter yield (*p* = 0.025), half-eviscerated yield (*p* = 0.038), and eviscerated yield (*p* = 0.020). Birds receiving 5 and 10 mg/kg irisin exhibited higher slaughter yields compared with the control group. Breast muscle yield was significantly affected (*p* = 0.004), with the highest value observed in the 5 mg/kg group (23.26%), followed by the 10 mg/kg group (22.78%), compared with the control group (21.39%).

### 3.3. Meat Quality

As shown in Table 4, dietary irisin supplementation significantly affected several meat quality parameters. In breast muscle, pH_45_ min was significantly influenced (*p* = 0.023). Cooking loss was markedly reduced by irisin supplementation (*p* < 0.001), with the lowest value observed in the 10 mg/kg group (19.20%) compared with the control group (27.77%). In leg muscle, L* value was significantly affected (*p* = 0.009). Drip loss was reduced in the 5 mg/kg group (*p* = 0.040). Cooking loss decreased progressively with increasing irisin supplementation (*p* < 0.001), reaching 29.78% in the 10 mg/kg group compared with 38.45% in the control group. No significant differences were observed among treatments for the remaining meat quality parameters (*p* > 0.05).

### 3.4. Serum Biochemical Parameters

**Figure 1:**
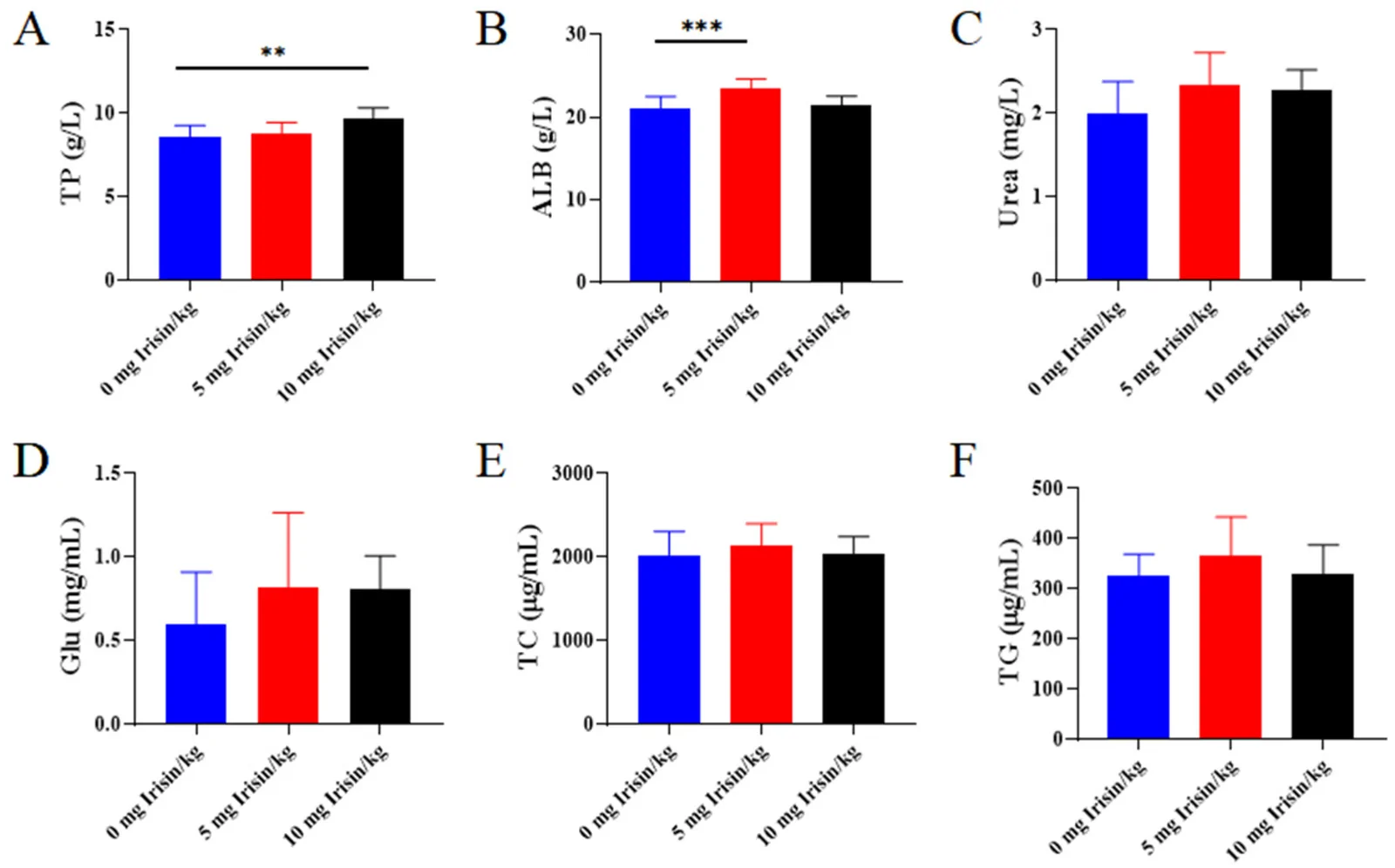
Effects of dietary irisin supplementation on serum biochemical parameters in broilers. (**A**) Total protein (TP), (**B**) albumin (ALB), (**C**) urea, (**D**) glucose (Glu), (**E**) total cholesterol (TC), and (**F**) triglycerides (TG). Broilers were fed diets supplemented with 0, 5, or 10 mg/kg recombinant chicken irisin for 42 d. Values are presented as means ± SEM. *n* = 6 birds per treatment, with one bird selected from each replicate pen. Differences among groups were analyzed using one-way ANOVA followed by Bonferroni-adjusted post hoc multiple comparisons. Asterisks indicate significant differences (** *p* < 0.01, *** *p* < 0.001).

The effects of dietary irisin supplementation on serum biochemical parameters at 42 days are shown in Figure 1. Serum total protein was significantly increased in broilers supplemented with 10 mg/kg irisin compared with the control group (Figure 1A). In addition, serum albumin concentration was significantly elevated in the 5 mg/kg group (Figure 1B). No significant differences were observed among treatments for serum urea, glucose, total cholesterol, or triglycerides (Figure 1C–F).

### 3.5. Antioxidant Indices

**Figure 2:**
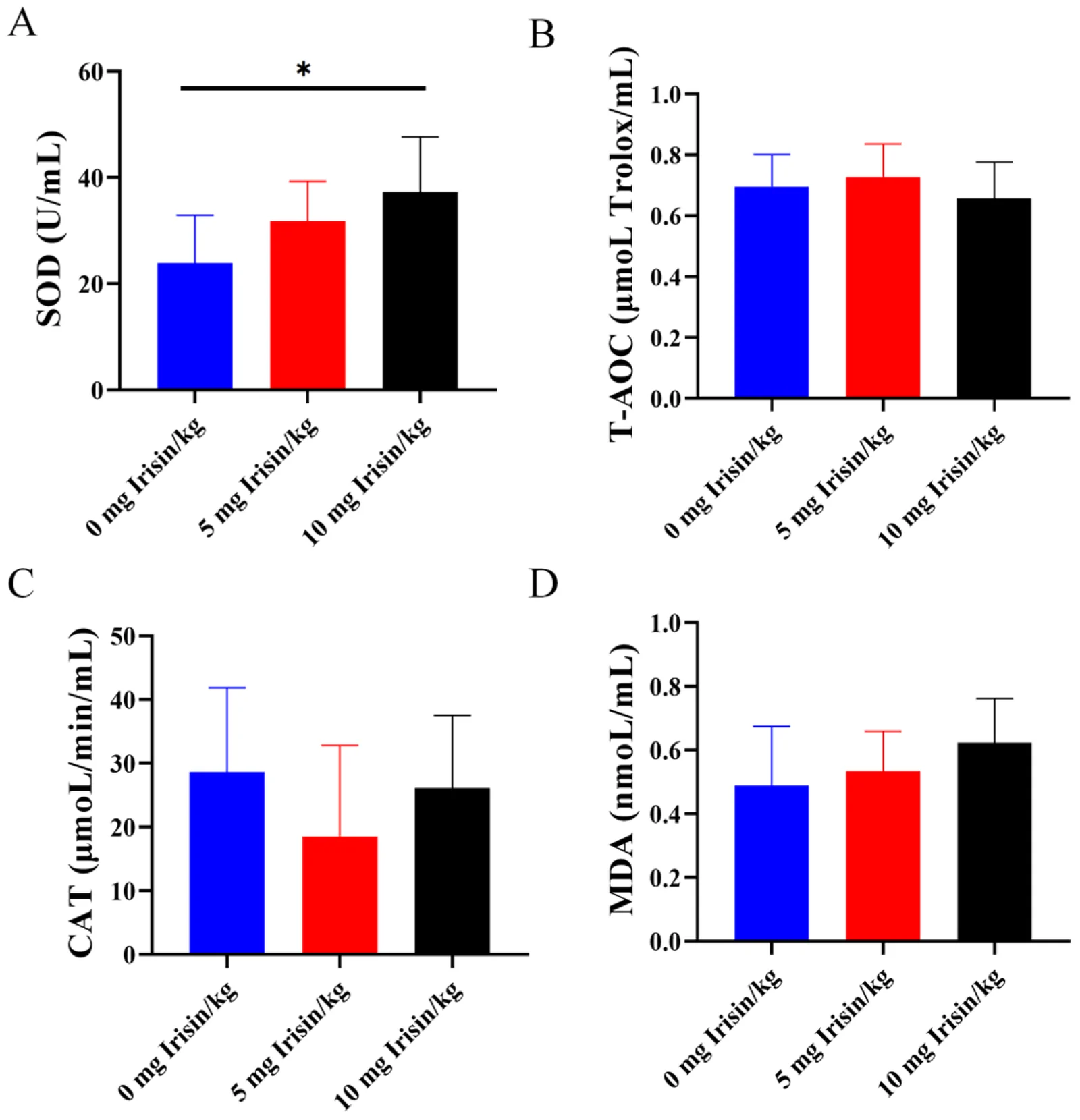
Effects of dietary irisin supplementation on serum antioxidant parameters in broilers. (**A**) Superoxide dismutase (SOD), (**B**) total antioxidant capacity (T-AOC), (**C**) catalase (CAT), and (**D**) malondialdehyde (MDA). Broilers were fed diets supplemented with 0, 5, or 10 mg/kg recombinant chicken irisin for 42 d. Values are presented as means ± SEM. *n* = 6 birds per treatment, with one bird selected from each replicate pen. Differences among groups were analyzed using one-way ANOVA followed by Bonferroni-adjusted post hoc multiple comparisons. Asterisks indicate significant differences (* *p* < 0.05).

The effects of dietary irisin supplementation on serum antioxidant status at 42 d are presented in Figure 2. Serum SOD activity was significantly increased in the 10 mg/kg group compared with the control group (Figure 2A). No significant differences were observed among treatments for T-AOC, CAT, or MDA levels (Figure 2B–D).

### 3.6. Immune Organ Indices

As shown in Table 5, dietary irisin supplementation had no significant effect on thymus, spleen, or bursa indices at 42 days of age (*p* > 0.05).

### 3.7. Serum Immunoglobulins and Cytokines

**Figure 3:**
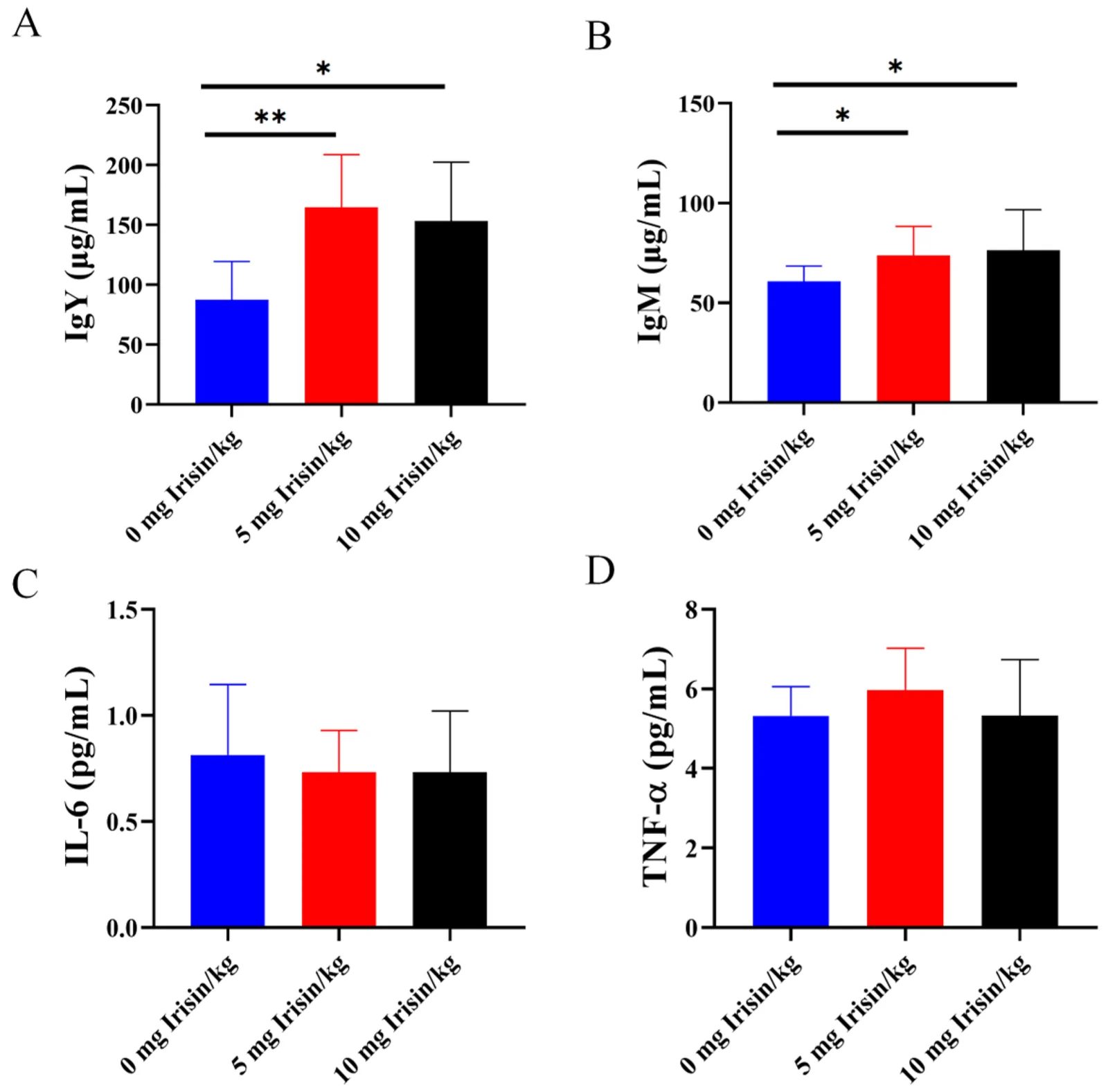
Effects of dietary irisin supplementation on serum immune parameters in broilers. (**A**) Immunoglobulin Y (IgY), (**B**) immunoglobulin M (IgM), (**C**) interleukin-6 (IL-6), and (**D**) tumor necrosis factor-α (TNF-α). Broilers were fed diets supplemented with 0, 5, or 10 mg/kg recombinant chicken irisin for 42 d. Values are presented as means ± SEM. *n* = 6 birds per treatment, with one bird selected from each replicate pen. Differences among groups were analyzed using one-way ANOVA followed by Bonferroni-adjusted post hoc multiple comparisons. Asterisks indicate significant differences (* *p* < 0.05, ** *p* < 0.01).

The effects of dietary irisin supplementation on serum immune-related parameters at 42 d are shown in Figure 3. Serum IgY concentration was significantly increased in both irisin-supplemented groups compared with the control group, with the highest value observed in the 5 mg/kg group (Figure 3A). Serum IgM concentration was also significantly elevated in the 5 and 10 mg/kg groups compared with the control group (Figure 3B). No significant differences were observed among treatments for serum IL-6 or TNF-α levels (Figure 3C,D).

### 3.8. Gut Microbiota Composition

**Figure 4:**
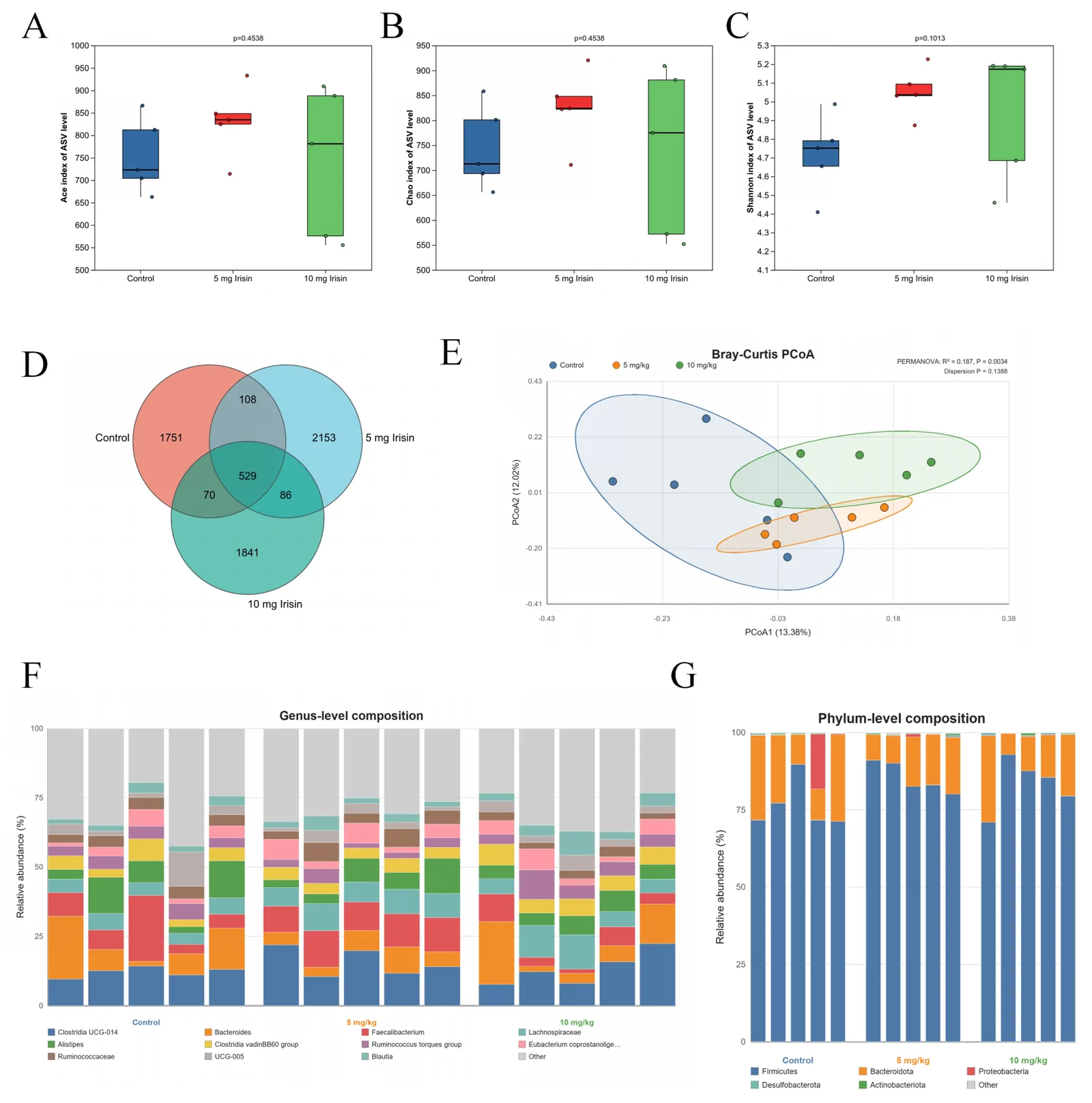
Effects of dietary irisin supplementation on cecal microbial diversity and composition in broilers. (**A**) Ace index, (**B**) Chao index, (**C**) Shannon index, (**D**) Venn diagram of shared and unique ASVs among treatment groups, (**E**) principal coordinate analysis based on Bray–Curtis distance, (**F**) relative abundance of dominant taxa at the genus or higher taxonomic levels, and (**G**) relative abundance of major phyla. Broilers were fed diets supplemented with 0, 5, or 10 mg/kg recombinant chicken irisin for 42 d. Five cecal-content samples per treatment were submitted for sequencing (*n* = 5 per treatment).

As shown in Figure 4, dietary irisin supplementation did not significantly affect the Ace, Chao, or Shannon indices (Figure 4A–C), indicating that irisin supplementation did not significantly alter microbial richness or alpha diversity under the present experimental conditions. Venn analysis showed that the control, 5 mg/kg, and 10 mg/kg groups contained 1751, 2153, and 1841 unique ASVs, respectively, with 529 ASVs shared among all groups (Figure 4D). Principal coordinate analysis based on Bray–Curtis distance showed a tendency toward separation among dietary treatments (Figure 4E). PERMANOVA confirmed significant differences in overall microbial community structure among groups (R^2^ = 0.187, *p* = 0.0034), whereas PERMDISP analysis showed no significant difference in within-group dispersion (*p* = 0.1388).

At the genus level, the cecal microbiota was mainly composed of Clostridia-related taxa, Bacteroides, Faecalibacterium, Lachnospiraceae-related taxa, Alistipes, Ruminococcus, Eubacterium, Blautia, and Lactobacillus (Figure 4F). Several genera showed variation in relative abundance among dietary treatments.

At the phylum level, the cecal microbiota was dominated by Firmicutes and Bacteroidota (Figure 4G). Variation in relative abundance was observed among treatments for major phyla, including Firmicutes, Bacteroidota, and Proteobacteria.

## 4. Discussion

Optimizing growth efficiency, carcass value, meat quality, and health resilience remains a central objective in modern broiler production. With the progressive restriction of antibiotic growth promoters and increasing consumer demand for high-quality poultry products, there is a growing need for functional feed additives capable of enhancing both productivity and physiological stability [2,17]. The present study demonstrates that dietary irisin supplementation at 5–10 mg/kg significantly improved growth performance, carcass yield, meat quality, antioxidant capacity, humoral immune response, and gut microbial structure in white-feathered broilers. Collectively, these findings indicate that irisin functions as a systemic metabolic regulator that coordinates nutrient utilization, redox homeostasis, immune competence, and intestinal ecological balance, thereby promoting production efficiency without compromising physiological equilibrium.

Feed efficiency is a major economic determinant in broiler production systems [18,19]. In the present study, dietary irisin supplementation increased final body weight and average daily gain while reducing feed conversion ratio. These concurrent responses indicate that irisin supplementation improved overall growth performance and feed efficiency in broilers. The increase in feed intake accompanied the enhanced growth response, while the lower FCR indicates that body-weight gain increased relative to feed consumption.

Moreover, improved redox homeostasis may mitigate oxidative damage-driven metabolic inefficiency. Oxidative stress is well recognized to compromise poultry performance by impairing mitochondrial bioenergetics, promoting macromolecular oxidation, and increasing maintenance energy expenditure, thereby reducing the proportion of dietary energy available for productive growth [20]. In skeletal muscle, excessive ROS can directly damage respiratory enzymes and membrane lipids, and it can also activate proteolytic/catabolic programs that oppose net protein accretion [21]. The elevated serum superoxide dismutase (SOD) activity observed at day 42 therefore supports an enhanced systemic antioxidant defense that may help preserve mitochondrial and cellular integrity during the peak growth phase [21]. Consistent with evidence from other species that irisin can engage antioxidant-regulatory networks and attenuate oxidative stress [9,10], irisin supplementation in broilers may create a redox-permissive metabolic milieu that favors efficient protein deposition while minimizing energy loss to oxidative damage and repair processes.

The increased slaughter yield and breast muscle proportion were further consistent with preferential lean tissue accretion. Breast muscle represents one of the most economically valuable components of the broiler carcass and is composed predominantly of fast-twitch glycolytic fibers characterized by rapid growth and marked metabolic plasticity [22,23]. Such characteristics make breast muscle highly responsive to changes in nutrient availability and metabolic signaling. Given the established involvement of irisin in skeletal muscle and energy metabolism, the greater breast muscle proportion observed in the supplemented birds may reflect altered nutrient partitioning or regulation of muscle growth. Notably, abdominal fat percentage was not significantly increased, suggesting that the improvement in carcass yield was not accompanied by greater abdominal fat deposition.

Beyond carcass traits, meat quality—particularly water-holding capacity—is an important determinant of processing yield and consumer acceptance [24,25,26,27]. In the present study, cooking loss was reduced in both breast and leg muscles, indicating improved water retention during thermal processing. In contrast, pH, color, drip loss, and shear force were largely unchanged, suggesting that the effect of irisin was relatively specific to thermal water-holding capacity rather than reflecting a broad alteration in postmortem muscle characteristics.

Effective immune function is important for maintaining health and productivity in intensive poultry production, where birds are continuously exposed to environmental and infectious challenges [28]. In the present study, dietary irisin supplementation increased serum IgY and IgM concentrations, indicating an effect on selected components of the humoral immune response, as circulating immunoglobulins are commonly used as indicators of humoral immune status in chickens and can respond to nutritional modulation [29,30]. Notably, these changes were not accompanied by increases in the measured pro-inflammatory cytokines IL-6 and TNF-α. This pattern suggests that the elevation in immunoglobulin concentrations occurred without detectable systemic activation of the inflammatory markers assessed in this study. Such a distinction may be physiologically relevant because sustained inflammatory signaling can increase maintenance energy expenditure and redirect nutrients away from productive processes such as growth [31]. Therefore, the concurrent increase in IgY and IgM and the absence of changes in circulating IL-6 and TNF-α may reflect a selective modulation of humoral immune status rather than a generalized inflammatory response.

The cecal microbiota plays important roles in nutrient metabolism, intestinal homeostasis, and immune regulation in poultry [32,33]. In the present study, dietary irisin supplementation did not significantly affect microbial richness or alpha diversity, whereas PERMANOVA revealed significant differences in overall community structure among treatments. The absence of a significant PERMDISP effect further indicated that these differences were not primarily driven by unequal within-group dispersion. At the taxonomic level, Firmicutes and Bacteroidota were the dominant phyla, while several major genera showed numerical variation among treatments. These findings suggest that irisin supplementation may reshape cecal microbial community composition without markedly altering overall diversity.

From a commercial perspective, supplementation at 5–10 mg/kg provided consistent benefits without detectable adverse effects. The combined improvements in feed efficiency, breast yield, meat quality, and immune status are economically meaningful. In antibiotic-reduced production systems, multifunctional additives capable of supporting both performance and health are increasingly valuable. However, practical implementation requires further validation. Cost-effectiveness, feed stability during pelleting, compatibility with existing nutritional strategies, and long-term safety assessments must be evaluated. Field-scale trials under commercial stress conditions will be essential to confirm reproducibility.

## 5. Conclusions

In summary, our results indicate that dietary irisin supplementation improved growth performance and selected carcass and meat-quality traits and was associated with changes in serum SOD activity, immunoglobulin concentrations, and cecal microbial composition. These findings support further investigation of recombinant irisin as a potential functional feed additive in broilers. However, further studies with larger experimental units and comprehensive validation approaches are required to establish the optimal dietary dose, evaluate the stability and bioavailability of dietary irisin, validate microbiota-associated responses, and elucidate the underlying biological mechanisms.

## Figures and Tables

**Table 1:** Ingredient composition and calculated nutrient levels of the basal diets (air-dry basis).

Item	Starter (1–21 d)	Grower (22–42 d)
***Ingredients (%)***		
Corn	55.24	59.37
Soybean meal	36.92	31.90
Soybean oil	3.50	5.00
Limestone (CaCO_3_)	1.12	1.23
Dicalcium phosphate (CaHPO_4_)	2.10	1.50
DL-Methionine	0.28	0.27
L-Lysine (98%)	0.22	0.11
Sodium chloride (NaCl)	0.30	0.30
Vitamin premix ^1^	0.03	0.03
Mineral premix ^2^	0.20	0.20
Choline chloride (70%)	0.09	0.09
Total	100.00	100.00
***Calculated nutrient levels* ^3^**		
Metabolizable energy (kcal/kg)	2950	3100
Crude protein (%)	21.00	19.00
Calcium (%)	1.00	0.90
Total phosphorus (%)	0.67	0.56
Non-phytate phosphorus (%)	0.45	0.35
Digestible lysine (%)	1.20	1.00
Digestible Met + Cys (%)	0.85	0.80
Digestible threonine (%)	0.66	0.60
Digestible tryptophan (%)	0.22	0.20

^1^ Vitamin premix supplied the following per kilogram of diet: Vitamin A, 12,000 IU; Vitamin D_3_, 3000 IU; Vitamin E, 30 mg; Vitamin K_3_, 3 mg; Vitamin B_1_, 2 mg; Vitamin B_2_, 8 mg; Vitamin B_6_, 4 mg; Vitamin B_12_, 0.02 mg; niacin, 40 mg; pantothenic acid, 15 mg; folic acid, 1.0 mg; biotin, 0.15 mg. ^2^ Mineral premix supplied the following per kilogram of diet: Fe (as ferrous sulfate), 80 mg; Cu (as copper sulfate), 10 mg; Mn (as manganese sulfate), 100 mg; I (as potassium iodide), 1.0 mg; Se (as sodium selenite), 0.30 mg. No separate zinc source or concentration was documented in the retained formulation records. ^3^ Nutrient levels were calculated values. Diets were formulated to meet or exceed the nutrient requirements of broiler chickens according to NRC (1994) [13].

**Table 2:** Effects of dietary irisin supplementation on overall growth performance of white-feathered broilers (1–42 d).

Items	0 mg/kg	5 mg/kg	10 mg/kg	SEM ^1^	*p*-Value
***Body weight (g)***					
1 d	39.42	39.83	39.50	0.133	0.592
21 d	628.35	622.87	628.67	1.518	0.533
42 d	2415.50 ^b^	2525.17 ^a^	2526.88 ^a^	15.688	0.017
***1–42 d***					
ADG (g/d)	56.57 ^b^	59.17 ^a^	59.22 ^a^	0.373	0.017
ADFI (g/d)	85.67 ^c^	86.33 ^b^	86.73 ^a^	0.103	<0.001
FCR	1.51 ^a^	1.46 ^b^	1.46 ^b^	0.009	0.029

^1^ SEM = standard error of the mean. Means within a row lacking a common superscript differ (*p* < 0.05); the first letter in the series (a) denotes the largest mean.

**Table 3:** Effects of dietary irisin supplementation on carcass traits of white-feathered broilers.

Items	0 mg/kg	5 mg/kg	10 mg/kg	SEM ^1^	*p*-Value
Slaughter yield (%)	84.26 ^b^	85.31 ^a^	85.27 ^a^	0.185	0.025
Half-eviscerated yield (%)	81.06 ^b^	82.39 ^a^	82.22 ^a^	0.194	0.038
Eviscerated yield (%)	69.96 ^b^	71.58 ^a^	71.16 ^a^	0.205	0.020
Breast muscle yield (%)	21.39 ^c^	23.26 ^a^	22.78 ^b^	0.213	0.004
Leg muscle yield (%)	8.05	7.89	7.82	0.061	0.402
Abdominal fat (%)	0.97	1.00	1.02	0.268	0.930

^1^ SEM = standard error of the mean. Means within a row lacking a common superscript differ (*p* < 0.05); the first letter in the series (a) denotes the largest mean.

**Table 4:** Effects of dietary irisin supplementation on meat quality of white-feathered broilers.

Muscle	Items	0 mg/kg	5 mg/kg	10 mg/kg	SEM ^1^	*p*-Value
Breast	pH_45_min	6.27 ^b^	6.24 ^b^	6.31 ^a^^b^	0.018	0.023
	pH_24_h	6.05	6.04	6.05	0.025	0.082
	L* value	53.55	53.14	54.20	0.310	0.633
	a* value	5.94	5.92	5.83	0.150	0.263
	b* value	12.58	11.22	11.27	0.291	0.319
	Shear force (N)	26.05	31.99	32.40	1.396	0.348
	Drip loss (%)	2.43	2.25	2.36	0.080	0.724
	Cooking loss (%)	27.77 ^a^	25.14 ^b^	19.20 ^c^	0.630	<0.001
Thigh	pH_45_min	6.52	6.41	6.40	0.021	0.157
	pH_24_h	6.36	6.42	6.36	0.016	0.618
	L* value	58.44 ^a^^b^	56.95 ^b^	59.91 ^a^	0.347	0.009
	a* value	4.98	4.57	4.53	0.125	0.420
	b* value	9.88	9.32	9.37	0.292	0.667
	Shear force (N)	14.05	16.19	17.79	0.788	0.239
	Drip loss (%)	3.16 ^a^	2.53 ^b^	3.11 ^a^	0.159	0.040
	Cooking loss (%)	38.45 ^a^	34.83 ^b^	29.78 ^c^	0.691	<0.001

^1^ SEM = standard error of the mean. Means within a row lacking a common superscript differ (*p* < 0.05); the first letter in the series (a) denotes the largest mean.

**Table 5:** Effects of dietary irisin supplementation on immune organ indices in white-feathered broilers at 42 days.

Items	0 mg/kg	5 mg/kg	10 mg/kg	SEM ^1^	*p*-Value
Thymus index ^2^	2.05	2.26	2.20	0.088	0.786
Spleen index ^2^	0.71	0.80	0.75	0.027	0.688
Bursa of Fabricius index ^2^	1.35	1.42	1.33	0.065	0.698

^1^ SEM = standard error of the mean. ^2^ Immune organ index = organ weight (g)/body weight (kg).

## Data Availability

The original contributions presented in this study are included in the article. Further inquiries can be directed to the corresponding author.
